# Redundancy of *p75NTR* neurotrophin receptor function in development, growth and fertility in the rat

**DOI:** 10.1007/s11248-024-00395-9

**Published:** 2024-07-09

**Authors:** Stephen Meek, Karamjit Singh-Dolt, Linda Sutherland, Matthew G. F. Sharp, Jorge Del-Pozo, David Walker, Tom Burdon

**Affiliations:** 1 The Roslin Institute, RDSVS, University of Edinburgh, Easter Bush Campus, Midlothian, EH25 9RG UK; 2 Bioresearch and Veterinary Services, University of Edinburgh, Chancellors Building, 49 Little France Crescent, Edinburgh, EH16 4SB UK; 3 The Royal Dick School of Veterinary Science, University of Edinburgh, Easter Bush Campus, Midlothian, EH25 9RG UK; 4 Present Address: Leiden University Medical Center, Leiden University, Leiden, The Netherlands; 5 Present Address: VPG Histology, 637 Gloucester Rd, Horfield, Bristol, BS7 0BJ UK

**Keywords:** Rat, p75NTR, Neurotrophin receptor

## Abstract

The p75NTR neurotrophin receptor has positive and negative roles regulating cell survival in the nervous system. Unambiguous interpretation of p75NTR function in vivo has been complicated, however, by residual expression of alternate forms of p75NTR protein in initial *p75NTR* knock-out mouse models. As rats are the preferred rodent for studying brain and behaviour, and to simplify interpretation of the knock-out phenotype, we report here the generation of a mutant rat devoid of the p75NTR protein. TALEN-mediated recombination in embryonic stem cells (ESCs) was used to flank exon 2 of *p75NTR* with Lox P sites and produce transgenic rats carrying either un-recombined floxed *p75NTR*^*Ex2-fl*^, or recombined, exon-2 deleted *p75NTR*^*Ex2-Δ*^ alleles. Crossing *p75NTR*^*Ex2-fl*^ rats with a *Cre*-deleter strain efficiently removed exon 2 in vivo. Excision of exon 2 causes a frameshift after p75NTR Gly23 and eliminated p75NTR protein expression. Rats lacking p75NTR were healthy, fertile, and histological analysis did not reveal significant changes in cellular density or overall structure in their brains. p75NTR function is therefore largely dispensable for normal development, growth and basal homeostasis in the rat. However, the availability of constitutive and conditional *p75NTR*^*Ex2-Δ*^ rats provides new opportunities to investigate specific roles of p75NTR upon injury and during tissue repair.

**Supplementary Information:**

The online version contains supplementary material available at 10.1007/s11248-024-00395-9.

## Introduction

The p75NTR receptor is a key mediator of neurotrophin signalling and has been ascribed a variety of roles in regulating the growth and maintenance of the nervous system (Dechant and Barde 2002; Lu et al. 2005; Boskovic et al. 2019). The founding member of the tumour necrosis factor receptor family, p75NTR is a single membrane spanning protein that lacks intrinsic catalytic activity but contains an intracellular death domain (Vilar 2017). Depending on its interacting partners p75NTR can either promote neuron outgrowth and survival, or induce cell death (Lu et al. 2005; Becker et al. 2018). In association with the neurotrophin Trk receptors, p75NTR forms a high affinity receptor for mature processed neurotrophins and promotes cell survival. By contrast, when linked to the sortilin receptor and bound by unprocessed proneurotrophin ligands, p75NTR induces apoptosis. When bound to the Nogo receptor, p75NTR mediates signalling downstream of myelin proteins which control cell growth and resolution of immune responses. Through these diverse interactions p75NTR is thought to influence the balance between cell growth, survival and death within the nervous system (Lu et al. 2005; Becker et al. 2018).

Elucidating the functional role of p75NTR in vivo has relied upon studies of a series of knock-out mice. Mice homozygous for the deletion of exon 3 are viable and fertile, but have impaired innervation of peripheral sensory neurons, and reduced response to the neurotrophin NGF (Lee et al. 1992). Subsequent studies, however, showed that mice harbouring the exon 3 deletion still express a short form of p75NTR, complicating conclusions regarding p75NTR function (Schack et al. 2001). Disruption of *p75NTR* exon 4 eliminated expression of both the long and short from receptors in mice, but also generated a cryptic protein containing just the death domain (Paul et al. 2004; Naumann et al. 2002). This likely contributed to the high mortality of pre and post-natal pups, and growth retardation of surviving *p75NTR* exon 4 disrupted mice. Indeed, more recently, *p75NTR* knock-out mice carrying a deletion of exons 4–6 encoding the transmembrane and cytoplasmic domains, lacked any p75NTR functional protein but were viable, reproduced normally and displayed only relatively subtle neurological defects (Bogenmann et al. 2011). This suggests that, in contrast to early reports and despite the involvement of p75NTR in several signalling pathways and cellular processes, p75NTR function might be largely redundant for normal development and growth in the mouse.

To evaluate p75NTR function in another mammal, and as a comparison with the phenotypes described in previous mouse studies, we generated knock-out rats lacking p75NTR receptor. The laboratory rat is a preferred animal model in studies of brain function and the nervous system, due to its larger brain size, greater intelligence and mental flexibility when compared with the mouse (Russell Lindsey and Baker 2006; Aitman et al. 2016). Until relatively recently gene targeting in the rat was not straightforward. However, the development of genuine germ line competent rat ESCs and the advent of *in ovo* gene editing using programmable nucleases have enabled the routine application of state-of-the-art genetic engineering techniques to the rat (Meek et al. 2017). A constitutive *p75NTR* knock-out rat generated by ZNF-directed *in ovo* gene editing is commercially available (https://insights.envigo.com/). By contrast, here we describe using ESCs to generate mutant rats that are either constitutively or conditionally deficient for p75NTR, providing new animal models with which to dissect the apparently contrasting functions of this enigmatic neurotrophin receptor.

## Materials and Methods

### ESC culture

Rat ESCs (line DAK31), derived from E4.5 rat blastocysts of the inbred pigmented rat strain Dark Agouti were kindly supplied by Professor Austin Smith (Blair et al. 2012). ESCs were maintained on irradiated OF1 or DR4 mouse fibroblasts in 2iL (N2B27 medium, 1 µM PD0325901 [PD], 3 µM CHIR99021 [CH], 1000 U/ml mouse LIF). Cells were passaged every 2–3 days using TVP (0.025% trypsin, 1% chicken serum, and 1 µM EDTA) and plated at a density of 0.5–1 × 10^5^/cm^2^. MEK (PD) and GSK3 (CH) inhibitors were supplied by Axon Medchem.

### BAC recombineering

The targeting vector was designed to introduce two loxP sites flanking exon 2 to generate a Cre recombination-mediated conditional knockout of the *p75NTR* gene. The targeting vector was constructed by BAC recombineering using the pSIM17 plasmid as previously described elsewhere (Liu et al. 2003; Sharan et al. 2009). Briefly, the pSIM17 plasmid was electroporated into E. coli cells containing the rat *p75NTR* BAC to facilitate subsequent recombineering steps. First, a single loxP site containing an EcoRI restriction site was introduced in intron 1, 393 bp upstream of exon 2. Next, a loxP-frt-PGK-EM7-Neo-frt cassette was introduced into intron 2301 bp downstream of exon 2. Finally, the targeting construct consisting of 1 kb homology arms with the loxP flanked exon 2 and frt-flanked neo selection cassette was retrieved into plasmid PL611. The targeting vector was designed so that the TALEN heterodimer binding sites would be disrupted following successful targeting thus preventing cutting of the targeted allele.

### TALEN construction

The P75NTR TALEN pair (left target—GCAGGTGTGAGGTTTG, right target—CTGGCCCCCTTGGCTAGG) were designed to cut within the spacer region (GGCCAGATTTCAGCTCCA) 393 bp upstream of exon 2, and result in a double strand break adjacent to the loxP insertion site in intron 1. All TALENs were designed using the TALE-NT software (https://tale-nt.cac.cornell.edu/node/add/talen) and assembled by Golden Gate cloning using methods described by Sakuma and co-workers (Sakuma et al. 2013). The TALENs were cloned into pCAG-T7 TALEN (Sangamo)-FokI-ELD-Destination and pCAG-T7-TALEN (Sangamo)-FokI-KKR-Destination expression plasmids.

### Gene targeting by homologous recombination

1 × 10^6^ rat ESCs were plated on to a 6-well plate of mitotically-inactivated mouse DR4 fibroblasts and transfected with 500 ng linearized targeting construct and 250 ng each of left and right TALEN expression plasmids using Lipofectamine LTX (6.75 µl LTX + 2.25 µl PLUS reagent, Invitrogen) according to manufacturer’s instructions. 48 h post-transfection cells were re-plated on to DR4 fibroblasts at a variety of densities ranging from 0.5–10 × 10^2^/cm^2^ in 2iL with 150 µg/ml G418 and fed every two days. Resistant colonies were picked between 9–12 days post-plating and expanded for PCR screening and cryopreservation.

### FLP-mediated excision of the neomycin selection cassette

Rat ESCs were transfected as described above with 1 µg pCAGGS-FLP-IP plasmid using Lipofectamine LTX. 48 h post-transfection cells were re-plated on to DR4 fibroblasts at 0.5–10 × 10^2^/cm^2^ in 2iL and fed every two days. Twelve colonies were picked 9–12 d post-plating and expanded.

### Genomic DNA

Cells and tissue were lysed in 500 µl lysis buffer (100 mM Tris pH8.0, 200 mM NaCl, 5 mM EDTA, 0.2% SDS) containing 50 µg proteinase K at 55 °C for at least 2 h. Genomic DNA was precipitated by adding 500 µl isopropanol to the lysate and inverting several times to mix. DNA was pelleted at 9000 g for 10 min and the pellet washed twice with 70% ethanol before air-drying and resuspending in nuclease-free water.

### Southern blot

Eight to ten micrograms of genomic DNA were digested with 40 units of *Eco*RI restriction enzyme overnight at 37 °C. The following morning a further 40 units of enzyme was added and incubated at 37 °C for an additional 5 h. The resulting DNA fragments were resolved on a 0.7% TAE agarose gel overnight at 25 V. The DNA fragments were UV-nicked prior to transfer to Hybond N + Nylon membrane (GE Healthcare, RPN203B) as described in the manufacturer’s instructions. Following transfer, the DNA was UV cross-linked on to the membrane. Probes were prepared by PCR amplification of the rat *p75NTR* sequence flanking the homology arms to generate a 1004 bp 5’ probe (forward primer TCGTTGCGTTAGTCTTCCTTC, reverse primer GCCTGCTCACTTCTTTTAGGA) and a 1498 bp 3’ probe (forward primer GGCCATTCTGTCCTTTGCCTC, reverse primer GAACCCACCTAGCTCTCAGTA). 25 ng of probe DNA was radioactively labelled with α–dCTP ^32^P using High Prime (Roche, 11 585 592 001), then hybridised to the membrane overnight at 65 °C in Church solution containing 10 μg/ml sonicated Herring Sperm DNA and 10 μg/ml tRNA. Non-specifically bound probe was removed by washing in 2 × SSC/0.1% (w/v) SDS at 65 °C. The membrane was exposed to Kodak Biomax MS film at − 80 °C.

### Genotyping

Two independent PCR reactions were performed using 150 ng of genomic DNA. The first reaction consisted of primers designed to flank exon 2 (intr1_F1–AAGGTCTGCCTGTCTAATCTGG, intr2_R1-CATTGATCTTGCAGCACGGAA) to amplify 1959, 2168 and 1260 bp from *p75NTR* wild-type, floxed *p75NTR*^*Ex2-fl*^ and the KO *p75NTR*^*Ex2−∆*^ alleles respectively. The second reaction utilized the same reverse primer and the forward primer (intr2_F1–GAGGTTGGTAGAAGAGCATAGC) located within the Cre-deleted region, 5’ of exon 2 to amplify 87 and 208 bp from wild-type and the floxed *p75NTR*^*Ex2-fl*^ alleles respectively. No product is amplified from the *p75NTR*^*Ex2-∆*^ allele in the second reaction because the 5’ forward primer site is deleted by Cre recombinase-mediated excision of exon 2. Both reactions were performed using NEB Q5 HotStart Taq Polymerase under the following conditions; 98 °C for 1 min, followed by 32 cycles of 98 °C for 10 s, 67 °C for 30 s and 72 °C for 2 min with a final extension of 72 °C for 10 min. Products were visualised with ethidium bromide on a 1% (for intr1_F1/ intr2_R1) or 2% (intr2_F1/ intr2_R1) TAE agarose gel.

### In vivo cre recombination

A homozygous *p75NTR*^*ex2-fl/fl*^ conditional floxed male rat was crossed with a heterozygous female rat carrying the CAG-NCre transgene (Wistar-Tg(CAG-Ncre) 81Jmsk rat strain #301 supplied by RRRC). Skin samples from euthanised 3-day old pups were processed for genomic DNA as described above. Genomic DNA was amplified using primers (forward primer Cre5—GCGGCATGGTGCAAGTTGAAT and reverse primer Cre3—CGTTCACCGGCATCAACGTTT) designed to amplify a 233 bp product from the Cre transgene. PCR reactions were performed as above and products visualised with ethidium bromide on a 2% TAE agarose gel.

### RNA/cDNA preparation

The brains of E13.5 rats were collected in RNALater Stabilisation Solution (ThermoFisher, #AM7020) prior to lysis. RNA was purified using the RNeasy miniprep kit (Qiagen, #74,104) according to the manufacturer’s instructions. The recommended on-column DNA digestion was performed and the RNA eluted using 35 µl nuclease-free water. 1 µg RNA was used to synthesise cDNA using the Multi-temp cDNA Synthesis kit (Agilent, #200,436). The final reaction volume was made up to 1 ml with nuclease free water and used for RT-qPCR analysis.

### RT-qPCR analysis

RT-qPCR analysis was performed using the Platinum SYBR Green QPCR kit (Invitrogen #11,744,100) containing 10 µl cDNA with forward and reverse primers that hybridised to *p75NTR* exons 5 and 6 respectively (forward primer GAGAAACTGCACAGCGACAG and reverse primer CTCTACCTCCTCACGCTTGG). The reaction was performed on a Stratagene Mx3000 real-time PCR machine under the following conditions; 95 °C for 2 min followed by 40 cycles of 95 °C for 15 s, then 60 °C for 30 s, with a final, melting curve cycle consisting of 95 °C for 1 min, 60 °C for 30 s and 95 °C for 15 s.

### Western blot analysis

The brains of E13.5 rats were lysed and 30 µg of protein combined with 5 µl 4 × LDS Sample Buffer (ThermoFisher, #NP0004) and 2 µl 10 × Sample Reducing Agent (ThermoFisher, #NP0007) in a final volume of 20 µl with water. Samples were heated at 70 °C for 10 min prior to loading and electrophoresis on a 10% Bis–Tris polyacrylamide gel (ThermoFisher, #NP0316BOX), followed by electroblotting on to a PVDF membrane. After blocking at room temperature for 1 h with Intercept (PBS) Blocking Buffer (LI-COR, #927–70,001) the membrane was probed with anti-p75NTR antibody diluted 1:4000 (Merck, #07–476) and anti-tubulinβ3 antibody diluted 1:500 (Biolegend, #MMS-435P) at 4 °C overnight. The Merk #07–476 anti-p75NTR polyclonal antibody was raised against the intracellular domain (274–425 aa) of rat p75 neurotrophin receptor. The membrane was then probed using the secondary antibodies IRDye680RD (Donkey anti-mouse, 1:5000, Li-Cor, #926–68,072) AND IRDye800W (Goat anti-rabbit, 1:5000, LI-COR, #926–32,211) at room temperature for 1 h. The membrane was analysed using the LI-COR Odyssey Infrared Imaging System.

### Histological analysis

Brains from the rats were fixed in 4% neutral buffered formalin. Fixed brains were step-sectioned at the approximate same level for each animal, and embedded in paraffin wax. Sections were stained with haematoxylin and eosin (HE). The histological sections were scanned using the NanoZoomer-XR system (Hamamatsu, Welwyn Garden City, Hertfordshire, UK) to create whole slide digital images. Representative brain areas of interest from *p75NTR*^*ex2-Δ/Δ*^ and *p75NTR*^*ex2-fl/fl*^ males which included cerebellum (white matter, molecular and granular layers), caudoputamen and thalamus, were manually delineated using the QuPath open-source software Bankhead et al. (2027). Total cell counts of the selected areas were obtained using automated nucleus detection, and the total area calculated to formulate a cell density for each region. Other organs routinely processed and examined included liver, lung, kidney, heart, skeletal muscle, adipose and haired skin, for which several background findings were noted, but no treatment-related effects were detected. All pathological and histological analysis was performed blinded to the genotype.

### Statistical analysis

*P75NTR* KO and CKO rats used for histological analysis were treated as independent sample sets based on genotype (n = 3). No significant difference was expected between sample sets therefore to determine the significance level an unpaired, two-tailed t-test with a 5% significance level was performed using Microsoft Excel software.

### Chimaera generation

Rat blastocysts at E4.5 days post-coitum were collected by noon on the day of injection and cultured for 2–3 h in KSOM embryo culture medium to ensure cavitation. Cells were disaggregated in TVP, pelleted in N2B27 and pre-plated on gelatin-coated tissue culture plastic in 2iL for 45–60 min. Non-attached cells were pelleted and resuspended in N2B27 containing 20 mM HEPES buffer then kept on ice prior to injection. Blastocycts were injected with 10–12 cells, then surgically transferred into the uteri of pseudopregnant Sprague Dawley rats.

## Results

### Generation of *p75NTR*^*Ex2−Δ*^ and *p75NTR*^*Ex2−fl*^ alleles in rat

**Fig. 1:**
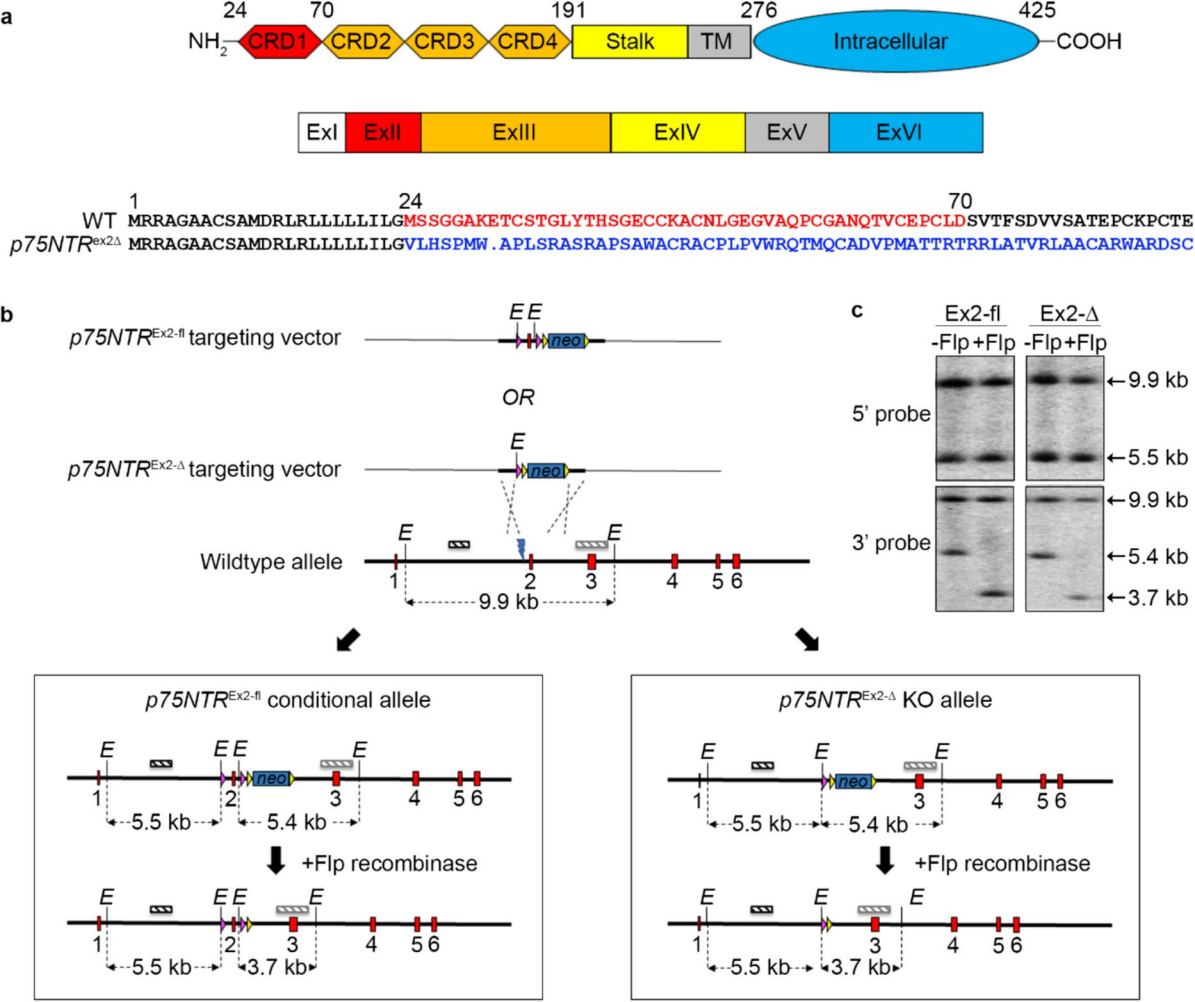
Generation of KO and conditional KO P75NTR rats. **a** The domain structure of p75NTR protein depicting the N-terminal extracellular domains consisting of four cysteine-rich domains (CRD1-4) and stalk region, the transmembrane domain (TM) and the intracellular C-terminal domain that contains the Chopper and Death domains (top schematic). The domains are encoded by six corresponding coding exons (middle schematic). Deletion of exon 2 (red) is predicted to generate an out-of-frame mutation at 24aa (blue sequence) and introduce a premature STOP codon at 31aa (blue dot) (bottom schematic). **b** Schematic of the *p75NTR* KO strategy showing the *p75NTR*^*Ex2-fl*^ and *p75NTR*^*Ex2-∆*^ targeting vectors, the wild-type *p75NTR* allele and the *p75NTR*^*Ex2-fl*^ and *p75NTR*^*Ex2-∆*^ alleles before and after FLP-mediated recombinase removal of the selection cassette. LoxP sites (pink arrowheads) flank exon 2, 408 bp 5’ and 325 bp 3’, with the FRT-flanked PGKneo selection cassette (blue box flanked by yellow arrowheads) 3’ of the 3’ loxp site. TALEN-mediated double-strand break (blue lightning bolt) was designed to promote homology-directed repair. Exons are depicted by red boxes, non-exonic genomic DNA and plasmid DNA by thick and thin black lines respectively. The EcoRI (E) restriction site and 5’ and 3’ probe sequences (dark and light hashed boxes respectively) used for Southern blot screening, and expected restriction digest fragments sizes are shown. **c** Southern blot analysis of EcoRI-digested genomic DNA from *p75NTR*^*Ex2-fl*^ and *p75NTR*^*Ex2-∆*^ targeted ESCs with (-Flp) and without (+ Flp) PGKneo selection cassette, using 5’ and 3’ external probes (top and bottom panels respectively). (Colour figure online)

We used targeted homologous recombination in ESCs to generate constitutive and conditional mutations in the rat *p75NTR* gene. Guided by a design strategy recommended by the EUCOMM mouse gene targeting consortium (https://www.mousephenotype.org/about-impc/about-ikmc/eucomm/) we elected to delete exon 2 of rat *p75NTR*, which results in a frameshift and the production of a 31 amino acid polypeptide containing only the first 23 amino acids of p75NTR (Fig. 1A). We generated a TALEN gene editing nuclease designed to introduce a double strand DNA break within intron 1393 bp upstream of exon 2; to promote the insertion of a neomycin resistance cassette nested by two Frt recombination sites, and flank exon 2 with two LoxP recombination sites (Fig. 1B). DAK31 rat ESCs (Blair et al. 2012) were electroporated with plasmids encoding the TALEN and one of two neomycin resistant targeting vectors, containing either a floxed exon 2 or the in vitro Cre-mediated recombinant deleted for exon 2. After 48 h recovery, the electroporated cultures were subjected to selection in 200 µg/ml G418 for a further 10 days. The resulting neomycin resistant rESC colonies were picked, expanded and screened by PCR and Southern Blotting for correct insertion of the targeting construct (Fig. 1C). Correctly targeted clones were transiently transfected with plasmid expressing FLP recombinase and subclones in which the neomycin cassette had been excised were selected based on sensitivity to neomycin. *p75NTR* exon 2 deletion subclones B10 and D10, and the floxed *p75NTR* subclone 34.4, having a normal karyotype and developmental potential as judged by embryoid body differentiation, were injected into Sprague Dawley (SD) blastocysts to generate ESC-derived chimaeric rats. Male chimaeric rats were crossed with SD females and germ line transmission of mutant *p75NTR* alleles through their offspring was used to establish transgenic lines of rats carrying either the *p75NTR* exon 2 deletion (*p75NTR*^*Ex2-Δ*^), or the floxed *p75NTR* (*p75NTR*^*Ex2-fl*^) allele (Table 1). Crossing *p75NTR*^*Ex2−Δ*^ heterozygous rats generated homozygotes that were viable and born at a normal Mendelian frequency (Table 2).

**Table 1:** Germline transmission efficiency

Cell line	Genomic modification	Chimaeras (sex)	GLT
34.4	*p75NTR*^ex2fl^	7 (6 M/1F)	4 (57%)
B10	*p75NTR*^ex2∆^	14 (14 M)	1 (7%)
D10	*p75NTR*^ex2∆^	6 (6 M)	2 (33%)

**Table 2:** Viability of P75NTR KO rats

Genotype	Number of offspring^a^ (% of total)	Sex
Wildtype	9 (18%)	4 M + 5F
Heterozygous	30 (59%)	11 M + 19F
Homozygous	12 (23%)	6 M + 6F

^**a**^Offspring from seven litters of a GLT x GLT mating

**Fig. 2:**
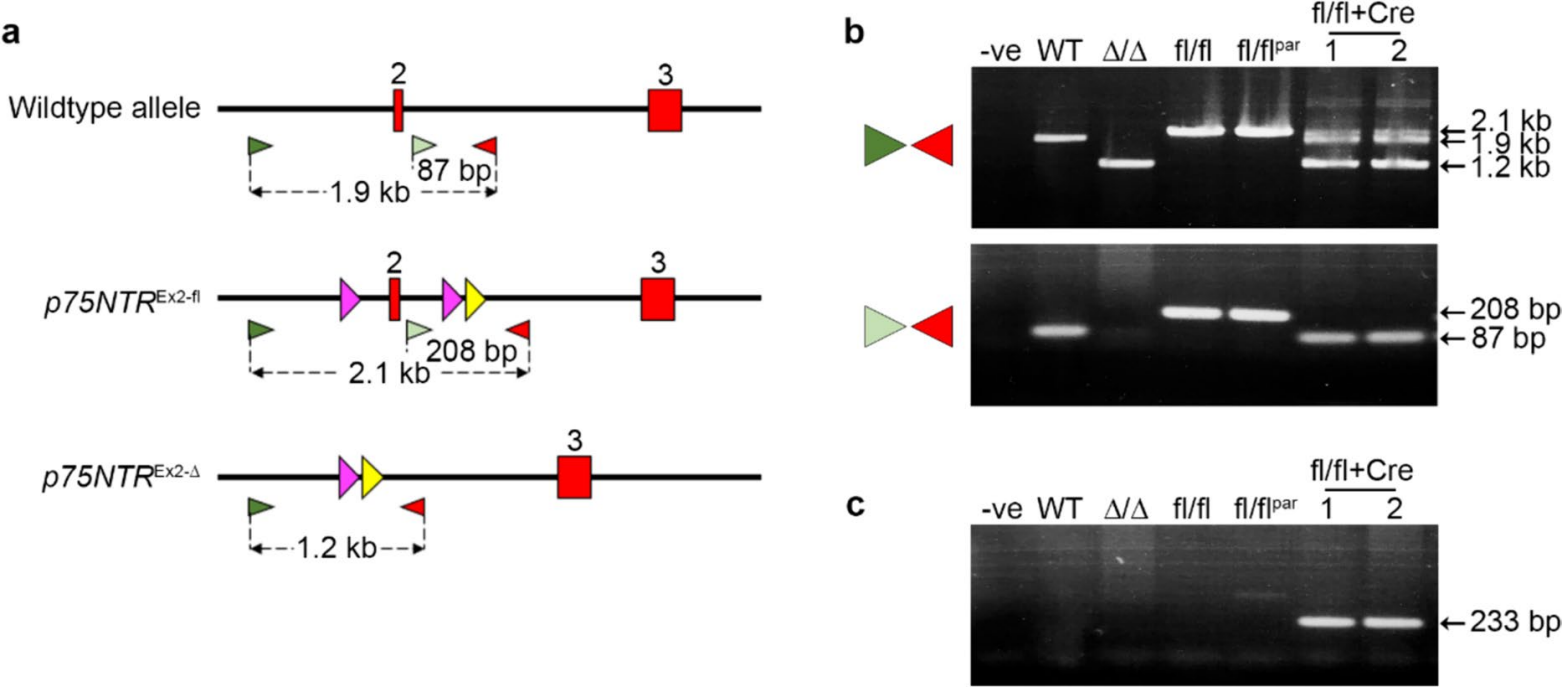
In vivo Cre recombinase-mediated deletion of conditional *p75NTR* allele. **a** PCR screening strategy used for detecting Cre recombinase-mediated deletion of exon 2 and discriminating between wild-type, *p75NTR*^*Ex2-fl*^ and *p75NTR*^*Ex2-∆*^ alleles. The locations of exons (red boxes), non-exonic genomic DNA (thick black lines), loxP sites (pink arrowheads), FRT sites (yellow arrowheads), forward PCR primers (light and dark green arrowheads) and reverse PCR primers (red arrowheads) are shown. Sizes of expected PCR fragments are highlighted. **b** PCR analysis of genomic DNA from wild-type rat ESCs (WT), and *p75NTR*^*Ex2-∆/∆*^* (∆/∆)* and *p75NTR*^*Ex2-l/fl*^* (fl/fl)* homozygous rats along with offspring (1 & 2) generated from breeding of a male homozygous *p75NTR*^*Ex2-fl/fl*^ (fl/flpar) with a female heterozygous Cre transgenic rat. The upper panel shows amplicons generated using the 5’ forward primer (dark green arrowhead) and reverse primer, demonstrating deletion of Exon 2 in *p75NTR*^*Ex2-fl/fl*^ /CRE offspring 1 & 2). The lower panel shows amplicons generated using the 3’ forward primer within the deleted region 5’ to exon 2 (lower panel) and the reverse primer. **c** PCR amplification of a 233 bp DNA fragment from the samples in 2b using primers specific for the CRE transgene. (Colour figure online)

To confirm that the *p75NTR* ^Ex2-fl^ allele could be efficiently recombined in vivo, male *p75NTR*^*Ex2-fl/fl*^ rats were bred with female *Cre*-deleter *CAG-NCre* transgenic rats (Sato et al. 2004). PCR analysis of resulting pups showed that exon 2 was efficiently deleted in all embryos by the Cre activity present within all the developing CAG-Cre oocytes (Fig. 2A–C) (Sakai and Miyazaki 1997).

### Deletion of *e*xon 2 eliminates p75NTR protein expression

**Fig. 3:**
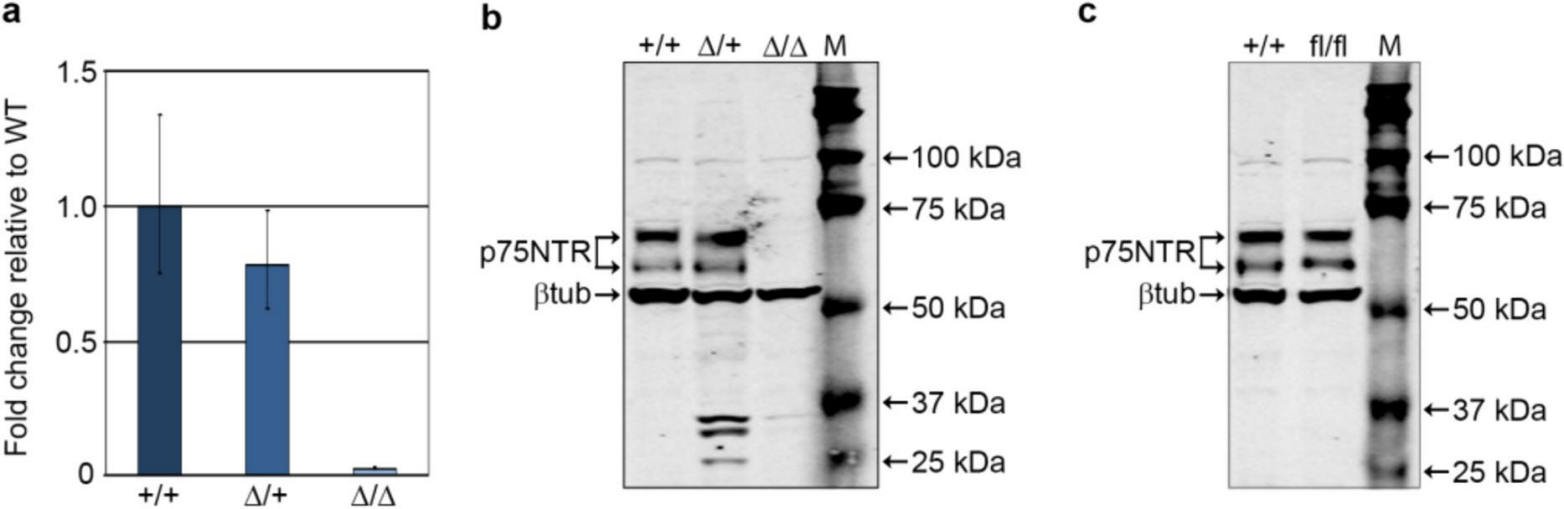
Loss of *p75NTR* expression in KO rats. **a** RT-quantitative PCR analysis of *p75NTR* in wildtype (+ / +), heterozygous (∆/ +) and homozygous (∆/∆) *p75NTR*^*Ex2-∆*^ rat brains (mean ± sd of three technical replicates). **b** Western blot analysis of p75NTR protein expression in wildtype (+ / +), heterozygous (∆/ +) and homozygous (∆/∆) *p75NTR*^*Ex2-∆*^ rat brains. **c** Western blot analysis of p75NTR protein expression in wildtype (+ / +), and homozygous (fl/fl) *p75NTR*^*Ex2-fl*^ rat brains. The images are composites of P75NTR and βtubulin exposures using the LICOR fluorescent detection system and bands located at 25-37 kDa were detected in the βtubulin channel

To determine how deletion of exon 2 affected p75NTR expression we crossed heterozygous *p75NTR*^*Ex2-Δ/*+^ rats and analysed brain tissue of their offspring. *p75NTR* RNA expression in *p75NTR*^*Ex2-Δ/*+^ heterozygotes was readily detected by RT-qPCR using primers annealing downstream of the exon 2, in exons 5 and 6. By contrast, the exon 2-deleted RNA was almost completely absent in homozygous *p75NTR*^*Ex2-Δ/Δ*^ rats suggesting that premature translation termination might destabilise the mutant RNA (Fig. 3A). Analysis of p75NTR protein expression in corresponding tissue lysates by Western blotting, using a polyclonal antibody recognising the intracellular domain of p75NTR, did not detect any p75NTR proteins in the brain of *p75NTR*^*Ex2-Δ/Δ*^ rats (Fig. 3B). By contrast, *p75NTR*^*Ex2-fl/fl*^ rats expressed p75NTR proteins at levels equivalent to that in wild-type *p75NTR* rats (Fig. 3C). We conclude that deletion of *p75NTR* exon 2 eliminates p75NTR protein, generating a functionally null allele, whilst in the floxed *p75NTR*^*Ex2-fl/ fl*^ rats, p75NTR expression was unaffected.

### Rats lacking p75NTR are healthy and fertile

p75NTR-deficient rats were visually indistinguishable from their p75NTR expressing littermates: they exhibited similar caged behaviour and possessed normal fertility. Indeed, mating p75NTR-deficient rats produced average sized litters of healthy pups (Table 3). As p75NTR-deficient mice have been reported to have deficits in neuronal development, we compared the histology of brains of *p75NTR*^*Ex2-Δ/Δ*^ and *p75NTR*^*Ex2-fl/fl*^ rats. Brain cellular density and structure in cerebellum, caudoputamen and thalamus regions was indistinguishable.

**Table 3:** Fertility of P75NTR KO rats

KO x KO	Litter size
Breeding 1	10 (5 M + 5F)
Breeding 2	7 (4 M + 3F)
Average litter size	8.5 (4.5 M + 4F)

between the p75NTR expressing and deficient rats (Fig. 4A, C and Supplementary Table S1). It was noted that brain size (weight) when normalised relative to body weight, was increased slightly in p75NTR-deficient rats (Fig. 4B). Other organs examined did not show this difference (Supplementary Table S2). Based on this general assessment, we conclude that under normal circumstances p75NTR has non-essential roles during development, growth and basal homeostasis of the adult rat.

**Fig. 4:**
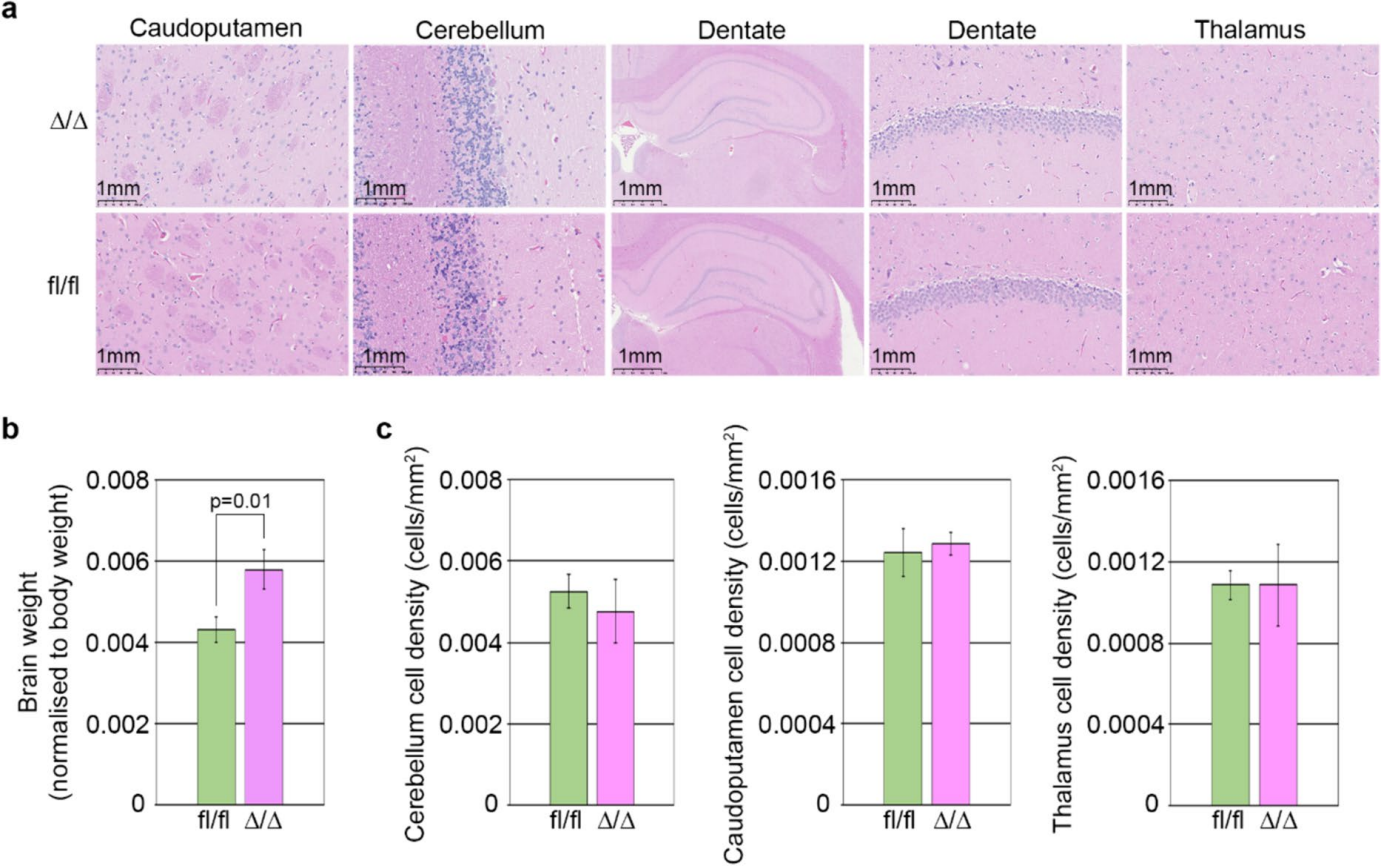
Histopathological analysis of *p75NTR*^*Ex2-∆/∆*^ and *p75NTR*^*Ex2-fl/fl*^ rats. **a** Representative images of haematoxylin and eosin-stained histological sections from male *p75NTR*^*Ex2-fl/fl*^ and *p75NTR*^*Ex2-∆/∆*^ homozygous rats. **b** Whole, fixed brain weight normalised to body weight, for *p75NTR*^*Ex2-fl/fl*^ and *p75NTR*^*Ex2-∆/∆*^ homozygous rats. The statistical analysis was performed using an unpaired two sample t-test with a 5% significance level (mean ± sd of three biological replicates). **c** Cell density in the cerebellum (left panel), caudoputamen (middle panel) and thalamus (right panel) of *p75NTR*^*Ex2-fl/fl*^ and *p75NTR*^*Ex2-∆/∆*^ homozygous rats (mean ± sd of three biological replicates)

## Discussion

The integration of neurotrophin-mediated cell survival and death signals by p75NTR is regarded as an important regulator of the fate of neurons and other cell types. However, the complexity of hypomorphic or gain–of-function mutations associated with the initial p75NTR transgenic studies in mice, upon which many early conclusions were based, can defy simple interpretation (Lee et al. 1992; Schack et al. 2001). By contrast, constitutive and conditional deletion of *p75NTR* exon 2 generates a complete null allele in the rat and secures a simple unambiguous experimental platform for exploring the function of p75NTR in vivo. Indeed, the viability and apparent health of the p75NTR-deficient rats prompts further examination of the role of p75NTR in neurotrophin signalling out-with normal development and basal homeostasis.

Deletion of *p75NTR* exon 2 eliminates expression of p75NTR protein in the rat. The mutation causes a translation frameshift that generates the first 23 amino acids of the N-terminal signal peptide of p75NTR (Johnson et al. 1986; Radeke et al. 1987), and an additional 7 unrelated amino acids. The mutation also suppresses expression of the *p75NTR*^*Ex2-Δ*^ transcript, presumably through a nonsense-mediated decay mechanism (Maquat 1995). Two different *p75NTR* mutant lines of mice are reported to completely eliminate p75NTR protein expression. Bogenmann and colleagues produced transgenic mice in which the region encompassing exons 4–6 is flanked by LoxP sites (*p75NTR*^*FX*^), thus allowing Cre-mediated deletion of the majority of the p75NTR intracellular domain, either constitutively or conditionally when crossed with suitable *Cre-*deleter strains of mice ^10^. By contrast, Boskovic and colleagues, inserted a LoxP recombination cassette in the exon1/intron 1 region (*p75NTR*^*Inv*^), that inverts its orientation through the action of Cre, replacing the coding region of Exon 1 with a mCherry reporter (Boskovic et al. 2014). Both the *p75NTR*^*FX*^ and *p75NTR*^*Inv*^ alleles enable the selective deletion of p75NTR from specific neuronal cells in mice. In a similar way selective deletion of rat p75NTR can be now achieved in rats carrying the *p75NTR*^*Ex2-fl*^ allele, either by crossing with Cre-driver strains, or by delivering Cre using viral vectors.

Given some of the phenotypes attributed to the loss of p75NTR in mice, we were surprised to find that p75NTR-deficient rats developed normally, were fertile and displayed no overt differences in growth or general behaviour from control rats. Although the mixed genetic background of the p75NTR-deficient rats (DA and Sprague Dawley) might mask subtle phenotypic changes in mutant animals when compared with p75NTR-expressing littermates, the roles of p75NTR in normal development and basal homeostasis appear to be non-essential in the rat. Interestingly, although p75NTR-deficient mice carrying a deletion of exons 4–6 were reported to exhibit minor signs of peripheral neuropathy, they otherwise appeared relatively normal and were fertile ^10^. The larger size of the brains of *p75NTR*^*Ex2-Δ*^ rats could hint that the normal pattern of neural development is disturbed by loss of p75NTR in rat. However, further experiments are required to eliminate differences that might arise from the slight age difference between the transgenic p75NTR rats in this study. Increases in the numbers of cholinergic neurons have been reported for *p75NTR* mutant mice (Naumann et al. 2002; Boskovic et al. 2014). Moreover, an increased size of the cerebellum was described in p75NTR mutant mice and was thought to result from over proliferation of cerebellar granule precursor cells (Zanin et al. 2016). p75NTR-deficient cerebellar cell cultures derived from a commercially available *p75NTR* knock-out rat (SAGE: https://insights.envigo.com/) also displayed a shortened cell cycle, but no associated effect on cerebellum or brain development was reported (Zanin et al. 2019). By contrast, conditional deletion of p75NTR in neural precursor cells of mice, using *Nestin*-driven Cre-mediated inactivation of the floxed *p75NTR*^*int*^ allele, was associated with a reduction in relative brain size and overall size of the mutant mice (Meier et al. 2019). Tissue-specific deletion of *p75NTR* in some cases has been reported to cause a more marked phenotype than constitutive deletion, implying that mechanisms induced early in development may compensate for loss of p75NTR (Zanin et al. 2016; Meier et al. 2019). Future experiments using acute, targeted, deletion of the conditional *p75NTR*^*Ex2−fl*^ allele, in conjunction with the corresponding constitutive null allele, should provide an opportunity to explore more precisely the role of p75NTR in the brain and other organ systems of the rat.

The larger size and intelligence of the rat, make this rodent the preferred experimental animal in many studies, particularly when investigating the brain and behaviour (Aitman et al. 2016). In addition, the rat has been used extensively as the standard model in pharmacology and toxicology studies. In this respect, the viability of the *p75NTR*^*Ex2-Δ*^ rat provides a simple, clearly defined, p75NTR-deficient animal model in which to definitively assess drug action or other experimental interventions. Since many reports implicate p75NTR in regulating responses to injury and regeneration (Schor 2005), the availability of the constitutive and conditional *p75NTR*^*Ex2-Δ*^ mutations make it feasible now to complement studies in mice, and assess how therapeutic strategies can be applied to ameliorate damage and promote repair, and determine their impact on behaviour and higher cognitive functions.

### Supplementary Information

Below is the link to the electronic supplementary material.

Supplementary file1 (XLSX 15 KB)

Supplementary file2 (XLSX 16 KB)

## Data Availability

The data underlying this report are available in the article (Supplementary Tables S1 and S2). Frozen embryos carrying the p75NTR cKO and KO alleles are available upon request.
